# Lineage Diversion of T Cell Receptor Transgenic Thymocytes Revealed by Lineage Fate Mapping

**DOI:** 10.1371/journal.pone.0001512

**Published:** 2008-01-30

**Authors:** Takeshi Egawa, Taras Kreslavsky, Dan R. Littman, Harald von Boehmer

**Affiliations:** 1 Molecular Pathogenesis Program, The Helen L. and Martin S. Kimmel Center for Biology and Medicine at the Skirball Institute for Biomolecular Medicine, New York University School of Medicine, New York, New York, United States of America; 2 Howard Hughes Medical Institute, The Helen L. and Martin S. Kimmel Center for Biology and Medicine at the Skirball Institute for Biomolecular Medicine, New York University School of Medicine, New York, New York, United States of America; 3 The Dana-Farber Cancer Institute, Harvard Medical School, Boston, Massachusetts, United States of America; Oklahoma Medical Research Foundation, United States of America

## Abstract

**Background:**

The binding of the T cell receptor (TCR) to major histocompatibility complex (MHC) molecules in the thymus determines fates of TCRαβ lymphocytes that subsequently home to secondary lymphoid tissue. TCR transgenic models have been used to study thymic selection and lineage commitment. Most TCR transgenic mice express the rearranged TCRαβ prematurely at the double negative stage and abnormal TCRαβ populations of T cells that are not easily detected in non-transgenic mice have been found in secondary lymphoid tissue of TCR transgenic mice.

**Methodology and Principal Findings:**

To determine developmental pathways of TCR-transgenic thymocytes, we used Cre-LoxP-mediated fate mapping and show here that premature expression of a transgenic TCRαβ diverts some developing thymocytes to a developmental pathway which resembles that of gamma delta cells. We found that most peripheral T cells with the HY-TCR in male mice have bypassed the RORγt-positive CD4^+^8^+^ (double positive, DP) stage to accumulate either as CD4^−^8^−^ (double negative, DN) or as CD8α^+^ T cells in lymph nodes or gut epithelium. Likewise, DN TCRαβ cells in lymphoid tissue of female mice were not derived from DP thymocytes.

**Conclusion:**

The results further support the hypothesis that the premature expression of the TCRαβ can divert DN thymocytes into gamma delta lineage cells.

## Introduction

“HY transgenic” mice express a transgenic T cell receptor (TCR) specific for HY antigen, i.e. a peptide of the Dby protein presented by class I D^b^ MHC molecules. In male transgenic mice, CD4^+^8^+^ double positive (DP) cells undergo negative selection by TCR agonist ligands and die by apoptosis [1]. However, CD8^+^ cells expressing mostly CD8α and low levels of CD8β as well as double negative (DN) cells with the transgenic TCR are present among extrathymic lymphocytes and intraepithelial gut lymphocytes (IEL) in male HY transgenic mice [2]. In fact, CD8^+^ cells with the transgenic TCR represent the most abundant population of T cells in the gut of these mice [3]. Two competing hypotheses with regard to the origin of these extrathymic cells have been put forward: one suggested that these cells survived the negative selection process at the DP stage of thymocyte development by down-regulating CD8 and CD4 co-receptor expression [4]. This hypothesis appeared consistent with later experiments showing that *in vitro* fetal thymic organ culture some DP cells from female HY-TCR transgenic mice could become CD8αα T cells when confronted with HY antigen [5]. Furthermore, our previous fate mapping experiments indicated that in wild-type (WT) mice CD8αα IEL with TCRαβ are derived from precursors expressing RORγt, which is an isoform of an orphan nuclear receptor, RORγ, encoded by the *Rorc* gene [6]. RORγt is specifically expressed in DP thymocytes. IEL with TCRγδ expression are not derived from RORγt expressing precursors. This led to the assumption that in WT and TCR transgenic mice CD8^+^ IEL are derived from DP thymocytes and that this pathway of differentiation represents induction of a lineage away from conventional TCRαβ cells that home to secondary lymphoid organs and towards intestinal intraepithelial TCRαβ lymphocytes that may have an important role in regulating gut immunity [7].

The competing hypothesis was that the CD8^+^ and DN cells expressing the HY transgenic TCR in male mice represent an abnormal subset of γδ lineage cells misguided by the premature expression of the transgenic TCRαβ that would mimic signals delivered normally by the TCRγδ. In addition, it was postulated that the confrontation of DN cells with the HY agonist ligand led to expression of the CD8α co-receptor as is observed with some activated TCRγδ cells [2]. Consistent with the second hypothesis was the observation that in mice exhibiting timely expression of the HY transgenic TCR, i.e. at the CD4^+^8^+^ stage of development, CD8αα IEL in the gut of male mice were much reduced [8] arguing that abnormally early expression of this particular transgenic TCR was indeed responsible for the generation of CD8^+^ IEL in male HY transgenic mice. Also, DN cells with the HY-TCR could be observed in the periphery of female mice indicating that a TCR agonist ligand was not required for their generation [1].

The former, but not the latter, hypothesis postulates that extrathymic lymphocytes with the HY receptor are derived from RORγt-positive DP precursors. By using RORγt-dependent lineage fate mapping in HY-TCR transgenic mice, it is therefore possible to determine which of the models provides an adequate explanation for how these cells are derived. RORγt is an orphan nuclear receptor, whose expression during T cell development begins in late DN cells and is mostly restricted to DP thymocytes [9], [10]. Lineage fate mapping using bacterial artificial chromosome (BAC) transgenic mice in which cre-recombinase is controlled by RORγt regulatory elements irreversibly labels cells developing through the conventional CD4^+^8^+^ TCRαβ, but not the DN TCRγδ, pathway. Moreover, IEL do not express RORγt [11]. Thus, breeding of the RORγt-cre mice to mice bearing a loxP flanked stop cassette in front of enhanced yellow fluorescent protein (EYFP) embedded in the *Rosa26* locus results in fluorescent labeling of all RORγt^+^ precursor-derived T cells [6], [12]. We showed that all TCRαβ cells in the secondary lymphoid organs of WT mice were labeled by RORγt-cre and that non-conventional TCRαβ populations of CD8ααTCRαβ IEL and Vα14i natural killer T (NKT) cells were likewise selected from a common pool of RORγt^+^ precursors [6], [13]. Utilizing this approach we have found that both of the original hypotheses concerning non-conventional TCRαβ cells in HY transgenic mice may be correct: the majority of intraepithelial CD8αα cells in the gut of male HY-TCR transgenic mice was derived from RORγt-negative precursors while a minority of HY-TCR^+^ CD8αα cells in male mice and all CD8αα cells in WT mice are derived from RORγt-positive precursor cells. Thus induction of the CD8ααTCRαβ lineage at the RORγt^+^ stage of development occurs in both WT and TCR transgenic mice whereas the precocious expression of a TCRαβ in TCR transgenic mice generates subsets of misguided TCRαβ-expressing T cells that are only rarely observed in normal mice.

## Results

### Origin of non-transgenic T cells with diverse αβ and γδTCRs in WT and TCR transgenic mice

**Figure 1:**
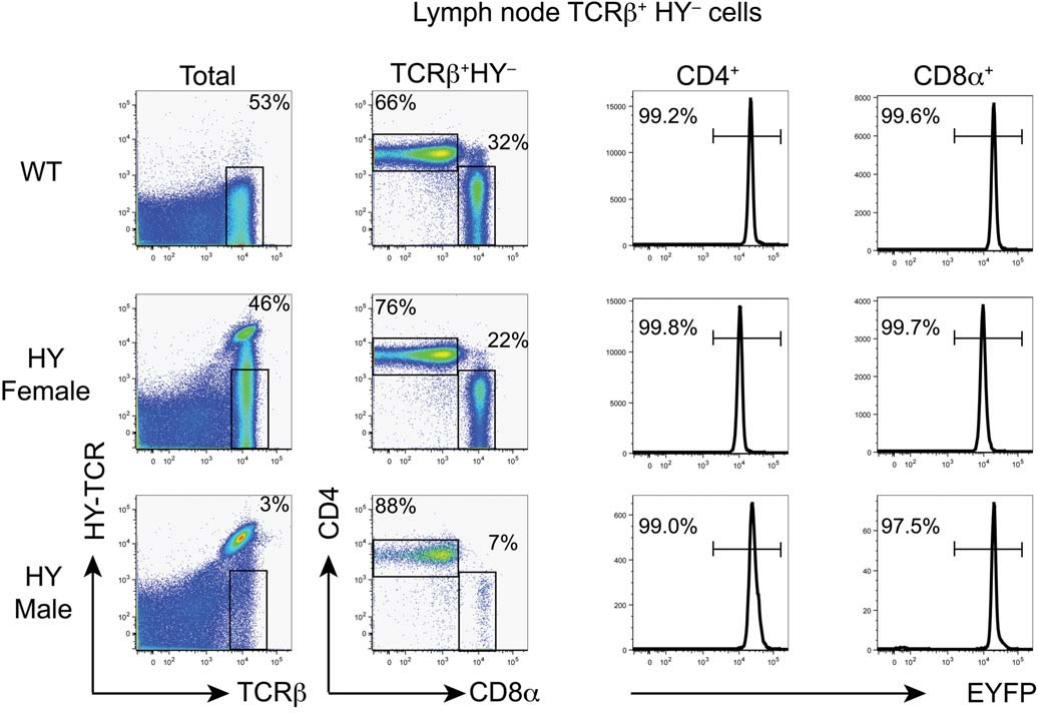
Fate mapping of T cells from WT and HY-TCR transgenic mice expressing endogenous TCRα using RORγt-cre. CD4 and CD8α expression by gated HY (T3.70)^−^ TCRβ^+^ lymph node cells (left column) is shown in the second column. EYFP expression by HY^−^CD4^+^ and HY^−^CD8α^+^ T cells is shown.

In RORγt-cre;ROSA26-stop-EYFP mice on a non-TCR transgenic background, practically all extrathymic cells with surface TCRβ chains were EYFP^+^ and thus derived from RORγt^+^ cells such as DP thymocytes (Figure 1). The same EYFP expression pattern was observed in extrathymic T cells from lymph nodes of male and female HY-TCR transgenic mice that did not express the transgenic TCR because of replacement of transgenic TCRα by endogenous TCRα chains (Figure 1) [14], [15]. This indicates that, after endogenous TCRα rearrangement in TCR transgenic mice, cells with new TCRs composed of transgenic TCRβ chain and endogenous TCRα chains develop in a similar pathway as they do in normal mice. Cells with the phenotype of extrathymic T cells were also found intrathymically (Figure S1) making it likely that they are all derived from RORγt^+^ DP thymocytes. Thus, in spite of the thymic cortex being largely devoid of DP thymocytes in male mice, some cells develop normally if they escape deletion by the early expression of endogenous TCRα chains.

**Figure 2:**
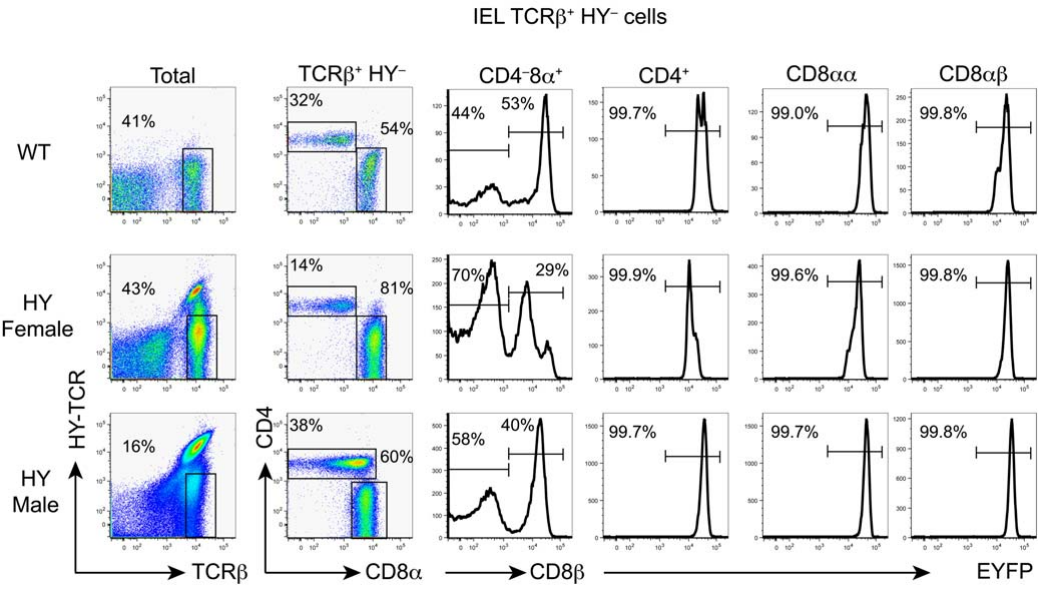
Fate mapping of IEL from WT and HY-TCR transgenic mice expressing endogenous TCRα using RORγt-cre. CD4 and CD8α expression by gated HY (T3.70)^−^ TCRβ^+^ IEL (left column) is shown in the second column. CD8β expression by gated CD8α^+^ cells (second column) is shown in the third column. EYFP expression by HY^−^CD4^+^, HY^−^CD8αα^+^ and HY^−^CD8αβ^+^ T cells is shown.

The pattern of reporter expression observed in IEL bearing endogenous TCRα chains was very similar to that obtained in lymph node T cells, with the exception that IEL contained a high proportion of CD8αα cells with TCRαβ that, like CD8αβ IEL, were EYFP^+^ (Figure 2). These results confirm earlier data on IEL in WT mice and in addition indicate that in TCR transgenic mice IEL with receptors other than the transgenic TCR are derived from RORγt^+^ precursors, presumably DP thymocytes. In contrast, the vast majority of IEL with TCRγδ was EYFP-negative in WT as well as in TCR transgenic mice (Figures 2 and S3). Thus, T cells with receptors other than the HY transgenic TCR, while they are relatively rare in TCR transgenic mice, develop along the same pathway as T cells in WT mice, indicating that normal pathways of T cell differentiation are operative in TCR transgenic mice.

### Origin of T cells with the transgenic HY TCR

**Figure 3:**
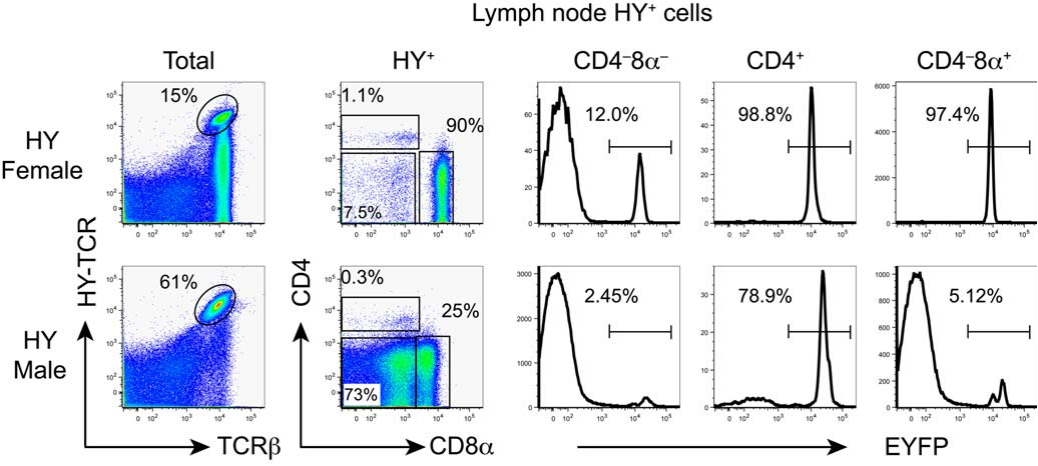
Fate mapping of lymph node T cells with the transgenic HY-TCR using RORγt-cre. CD4 and CD8α expression by gated HY (T3.70)^+^ lymph node cells (left column) is shown in the second column. EYFP expression by HY^+^CD4^−^CD8α^−^, HY^+^CD4^+^CD8α^−^ and HY^+^CD4^−^CD8α^+^ T cells is shown.

In female HY TCR transgenic mice, single positive thymocytes and lymph node T cells that express the transgenic TCR and either CD4 or CD8 co-receptor were all EYFP-positive and thus derived from DP thymocytes (Figures 3 and S2). The CD4^+^ subset includes cells with relatively high levels of the transgenic TCR. However, the generation of these cells requires co-expression of endogenous TCRα chains since such CD4^+^8^−^ T cells are absent in female HY TCR transgenic mice on the RAG-2^−/−^ background [16]. In contrast to WT mice, there was a substantial number of DN cells that expressed TCRαβ in female transgenic mice. The vast majority of these cells was EYFP-negative and thus was not derived from DP thymocytes. TCRαβ-expressing DN cells were also detected intrathymically (Figure S2). In male HY-TCR transgenic mice, T cells with the transgenic TCR exhibited an even more abnormal phenotypic pattern: the vast majority of DN cells and CD8^+^ cells, the latter expressing relatively low levels of CD8β, were EYFP-negative and hence were not derived from DP thymocytes.

**Figure 4:**
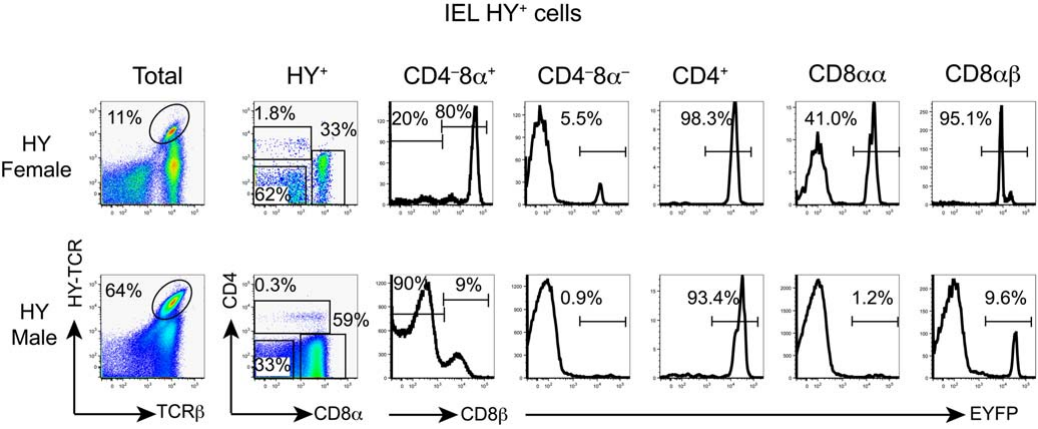
Fate mapping of IEL T cells with the transgenic HY-TCR using RORγt-cre. CD4 and CD8α expression by gated HY (T3.70)^+^ IEL (left column) is shown in the second column. CD8β expression by gated CD8α^+^ cells (second column) is shown in the third column. EYFP expression by HY^+^CD4^−^CD8α^−^, HY^+^CD4^+^, HY^+^CD8αα^+^ and HY^+^CD8αβ^+^ T cells is shown.

Similar abnormalities were noted in IEL, among which DN cells comprised a much larger compartment in female HY-TCR transgenic compared to WT mice (Figure 4); the majority of DN cells with the transgenic TCR did not develop through RORγt-expressing DP thymocytes, similar to most CD8αα cells that were likewise mostly EYFP-negative. However, in female mice, a substantial number of EYFP-positive cells was also present among HY^+^ IEL that were either DN or CD8αα suggesting that induction of these atypical lineages could have occurred at the DP stage. In contrast, all CD8αβ-positive cells with the transgenic TCR were RORγt^+^ cell-derived. In male HY-TCR transgenic mice, practically all DN, CD8αα and a majority of CD8αβ cells were EYFP-negative and hence were not derived from RORγt^+^ DP thymocytes. This was especially true for the exaggerated number of CD8αα IEL that in WT mice were all derived from RORγt^+^ precursors (Figures 2 and 4). The notion that most CD8α^+^ HY-TCR-expressing cells in IEL have a different origin than CD8α^+^ IEL of WT mice is supported by the finding that only the former expressed uniformly low levels of CD5 (data not shown).

**Figure 5:**
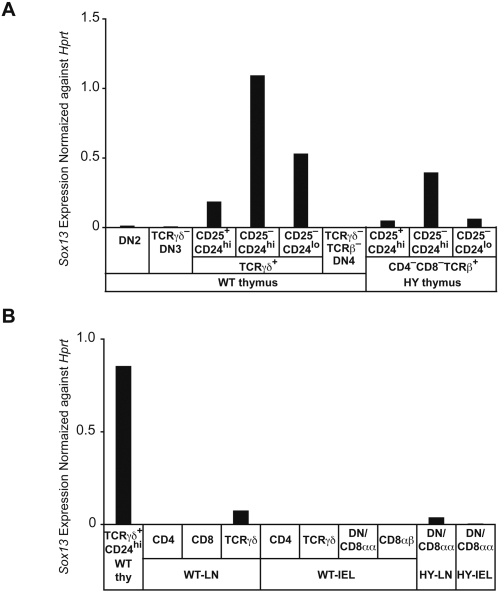
Sox13 expression in thymocyte subpopulations from HY transgenic mice. (A) *Sox13* expression in male HY^+^ DN thymocyte subpopulations, WT DN thymocyte subpopulations and TCRγδ^+^ thymocyte subsets was determined by real time PCR. (B) *Sox13* expression in peripheral T lymphocytes from WT and male HY transgenic mice. As a reference, *Sox13* expression in TCRγδ^+^ CD24^hi^ thymocytes, which was detected in the same real time PCR measurement, is shown together with *Sox13* expression in peripheral lymphocyte populations. Relative *Sox13* expression levels normalized against *Hprt* expression are shown. The data shown are representative from two experiments with similar results.

Initial studies suggested that the abnormal DN and CD8^+^ cells with the HY transgenic TCR have similarities with gamma delta lineage cells because, in contrast to conventional TCRαβ cells, they had retained the germline TCRδ (*Tcrd*) locus [17]. To further determine whether premature TCRαβ signals could induce a signature gene for the TCRγδ lineage, we purified thymic subpopulations from HY-TCR transgenic mice or WT mice and examined expression of Sox13, which has been shown to contribute to the development of TCRγδ cells [18] (Figure 5). We could observe upregulation of Sox13 in TCRγδ^+^CD24^+^CD25^+^ thymocytes as compared to DN2 thymocytes or TCRγδ^−^ DN3 thymocytes, and Sox13 was further upregulated in TCRγδ^+^CD24^+^CD25^−^ thymocytes (Figure 5A). However, Sox13 expression was then downregulated in TCRγδ^+^CD24^lo^ thymocytes and it became almost undetectable in TCRγδ^+^ IEL, while Sox13 expression was barely detected in TCRγδ^−^ DN4 or DP thymocytes which are selected from DN3 precursors by pre-TCR signals (Figure 5A and 5B; data not shown). These results suggest that Sox13 is transiently upregulated following signals through TCRγδ, but not pre-TCR, at the DN thymocyte stage and may play some role in specification of the TCRγδ lineage [18]. In thymocytes from male HY mice, we could observe significant upregulation of Sox13 in HY^+^DN thymocytes. Similar to WT TCRγδ lineage cells, Sox13 upregulation was detected at the CD25^+^CD24^+^ stage and peaked at the CD25^−^CD24^+^ stage followed by Sox13 downregulation as cells matured. These findings strongly support our hypothesis that premature TCRαβ signals divert a proportion of TCRαβ^+^ DN thymocytes into the TCRγδ lineage.

## Discussion

Most TCR transgenic mice express the transgenic TCRαβ at the DN stage of T cell development and thus earlier than the endogenous TCR in non-transgenic mice. This is the case even when normal regulatory elements of the *Tcra* and *Tcrb* genes have been used, such as in HY transgenic mice; in these mice, cosmids containing rearranged TCR genes were introduced and TCRβ and TCRα transgenes were integrated in tandem such that expression of the TCRα transgenes can be regulated by *Tcrb* control elements. Because of the premature TCRαβ expression, the validity of conclusions obtained in TCR transgenic mice has sometimes been questioned. A good example is the deletion of DP thymocytes in HY transgenic mice, whose physiological relevance was questioned by either assuming that the virtual absence of DP thymocytes in male HY-TCR transgenic mice was not caused by deletion but by a developmental arrest at the DN stage [19] or by arguing that the too early TCR expression in the DP compartment was responsible for deletion that normally would only occur in the medulla [8]. One of us has previously explained why these assumptions are unlikely to be true: DP cells are observed in the thymus of male HY-TCR transgenic mice and are increased in mice that lack CD8β co-receptor chains [20], [21], arguing against a developmental arrest at the DN stage as the sole explanation for the low proportion of DP thymocytes in these mice. These studies, together with another study using transgenic mice expressing a mutant D^b^ molecule [21], suggest that interaction of MHC class I and the CD8αβ/TCRαβ complex is important for efficient depletion of DP thymocytes in male HY mice. DP thymocyte deletion also occurs in male HY-TCR transgenic mice with timely onset of TCR transgene expression at the DP stage [8]. In addition, studies in WT and TCR transgenic mice show that ligation of the TCR on DP thymocytes results in their deletion [22]. Thus, there is convincing evidence that DP thymocytes are generally susceptible to deletion depending on the availability of appropriately presented antigen in the thymic cortex, especially class I MHC presented peptides derived from proteins such as HY antigen [23], and that deletion of DP thymocytes in TCR transgenic mice is representative of this physiological process.

The above considerations are important for the results presented here since it is believed that confrontation of DP thymocytes with cognate TCR ligands can result not only in deletion, but also in induction of development of alternative lineages, thereby generating T cells that can migrate from the thymus and regulate immunity in the gut as CD8αα T cells [7]. Since these conclusions were reached from studies that included experiments with TCR transgenic mice [5], careful consideration has to be given to the possibility that results may have been influenced by precocious expression of the TCRαβ in TCR transgenic mice. With regard to this issue, it is important to consider that in male HY transgenic mice CD8^+^ T cells with the transgenic TCR are present in peripheral lymphoid tissues and in IEL, in spite of the fact that the cortex is largely devoid of DP thymocytes. Two different scenarios were proposed to account for these observations: in one scenario, down-modulation of CD8β co-receptor at the DP stage was held responsible for the escape of T cells into the periphery [4] while in the other it was assumed that the CD8^+^ cells were generated through activation of a differentiation program for DN γδ lineage cells that express the transgenic TCRαβ, resulting in increased levels of CD8α [2].

The different hypotheses can be tested by using lineage fate mapping that can distinguish whether subsets of T cells develop through the RORγt^+^ stage or are derived directly from RORγt-negative precursors. The lineage fate mapping method utilized here had previously been employed to trace the origin of CD8αα cells in the gut and invariant NKT cells [6], [13]. Using the same approach in female and male HY-TCR transgenic mice, we have now delineated the origin of various peripheral T cells in lymph node and gut epithelium. The results do in fact show that the vast majority of lymph node T cells and IEL with the HY transgenic TCR and low level or no expression of CD8 co-receptors are lineage diverted most likely at the DN stage of thymocyte development before expression of RORγt sets in, and are thus independent of the DP thymocyte differentiation pathway. This hypothesis is also supported by studies using CD8β deficient mice or mice with a mutant MHC class I molecule [20], [21]. Although effective depletion of DP thymocytes relies on interaction of CD8αβ and the MHC class I molecule, accumulation of HY^+^ DN thymocytes was observed in CD8β deficient mice or in transgenic mice bearing a mutant D^b^ molecule, in which substantially large numbers of DP cells escape from deletion. These results suggest that the lineage diversion of HY^+^ DN cells does not require CD8αβ/MHC interaction and may take place independently of the DP stage in which the majority of HY^+^ cells are deleted. This conclusion applies to most, but not all, of the CD8αα cells in IEL, especially in male mice. In TCR transgenic mice, these cells are dependent on the premature expression of the transgenic TCRαβ in DN thymocytes and hence represent an artificially exaggerated population of cells that exists at a very low frequency in normal mice [24].

The few EYFP-labeled CD8αα cells with the transgenic TCR in male HY transgenic mice may be generated through the DP thymocyte stage, similarly to non-transgenic CD8ααTCRαβ IEL [5]. There is, however, a caveat to the assumption that these cells develop through the confrontation with TCR agonist ligands at the DP stage: EYFP-positive CD8αα cells with relatively high levels of the HY transgenic TCR are also found among IEL in female transgenic mice where the HY ligand is not present. However, it cannot be excluded that these cells also express endogenous TCR chains and hence may have encountered TCR agonist ligands intrathymically. The expression of endogenous TCRα chains cannot, however, explain the existence of EYFP-negative CD8αα IEL in female mice, since endogenous TCRα expression at the DN thymocyte stage is a rather rare event. These cells may express CD8α independently of cognate TCR ligands, perhaps facilitated by the gut microenvironment. We also do not completely rule out the possibility that a few EYFP-positive HY^+^ cells or EYFP^+^TCRγδ cells might turn on RORγt expression in the lamina propria, where some T cells express RORγt [11], and then migrate to the epithelium.

It is of interest to note that all extrathymic T cells in TCR transgenic mice that do not express the transgenic TCR exhibit the same EYFP labeling pattern as extrathymic T cells in WT mice, i.e. the TCR transgenes do not lead to major changes in lymphoid organ structure that prevent normal lymphocyte development.

In summary these studies suggest that consequences of premature TCRαβ expression have to be considered when analyzing pathways of lymphocyte subset generation. It had previously been argued that signals from the prematurely expressed TCRαβ may “fool” DN cells to develop into γδ lineage cells, including DN and CD8^+^ cells in lymph nodes and gut [2]. The lineage fate mapping studies reported here support this scenario. Our conclusion is further strengthened by our data demonstrating that Sox13 was upregulated in HY^+^ DN thymocytes following premature signals through TCRαβ instead of pre-TCR, as is by other reports showing that these abnormal cells exhibit profiles of surface marker and cytokine expression, which resemble those of TCRγδ cells rather than those of conventional TCRαβ cells [18], [25].

## Materials and Methods

### Mice

HY TCR transgenic mice [1] were either from the colony maintained in the Dana Farber Cancer Institute or purchased from Taconic. RORγt-cre and ROSA26-stop-EYFP mice were previously described [6], [12]. Mice were maintained in specific pathogen-free animal facilities at NYU School of Medicine, the Memorial Sloan-Kettering Cancer Center, or the Dana Farber Cancer Institute. Experiments were performed according to the protocols approved by the Institutional Animal Care and Use Committee at the individual institutes listed above.

### Flow Cytometric Analysis

Intraepithelial lymphocytes were prepared by exposing gut mucosa to 1 mM DTT in PBS with vigorous shaking for 10 minutes at room temperature. Staining of surface antigens was performed as described [13]. Antibodies except for anti-HY TCR (T3.70) were purchased from eBioscience (San Diego, CA). Anti-HY TCR antibody was provided by eBioscience. Dead cells were excluded by DAPI staining. Data were collected with an LSRII cytometer (BD Biosciences, San Jose, CA) using FACSDiva software and were analyzed with Flowjo software (Tree Star Inc., Ashland, OR).

### Real Time PCR Analysis

Thymocyte subsets and peripheral lymphocytes were sorted from WT and HY TCR transgenic mice in the *Rag2*-deficient background using FACSAria (BD Biosciences). Total RNA was prepared using the RNeasy kit (Qiagen) followed by DNase digestion (Qiagen). cDNA was synthesized using Superscript II and Superscript III reverse transcriptases by oligo(dT) priming (Invitrogen Life Technologies) according to the manufacturer's recommendations. Amounts of *Sox13* and *Hprt* RNA were quantified by real time PCR with SYBR Green. The primer sequences for *Sox13* were obtained from SuperArray (catalogue number PPM04782A-200). *Hprt* primers were described previously [26].

## Supporting Information

Figure S1 Fate-mapping of thymocytes from WT and HY-TCR transgenic mice expressing endogenous TCRα using RORγt. CD4 and CD8α expression by gated HY(T3.70)^−^ TCRβ^hi^ thymocytes (left column) is shown in the second column. EYFP expression by HY^−^CD4^−^8α^−^, CD4^+^8α^+^, CD4^+^8α^−^ and CD4^−^8α^+^subsets is shown. (There were very few, if any, HY^−^ TCRβ^hi^ CD4^−^8^−^ cells in the thymus of HY transgenic female or male mice.)
(3.03 MB TIF)

Figure S2 Fate mapping of thymocytes with the transgenic HY-TCR using RORγt-cre induced EYFP labeling. CD4 and CD8α expression by gated HY(T3.70)^hi^ thymocytes (left column) is shown in the second column. EYFP expression by CD4^−^8α^−^, CD4^+^8α^+^, CD4^+^8α^−^ and CD4^−^8α^+^ subsets is shown.
(2.34 MB TIF)

Figure S3 EYFP expression in TCRγδ IEL from RORγt-cre; ROSA26-stop-EYFP mice. TCRγδ positive IEL from WT and male and female HY-TCR transgenic mice were gated (top) and EYFP expression is shown (bottom).
(0.69 MB TIF)
