# Supplementary figures and images for: Lineage Diversion of T Cell Receptor Transgenic Thymocytes Revealed by Lineage Fate Mapping
Source: PLoS One. 2008 Jan 30;3(1):e1512. doi: 10.1371/journal.pone.0001512 (PMC2211402; doi:10.1371/journal.pone.0001512)

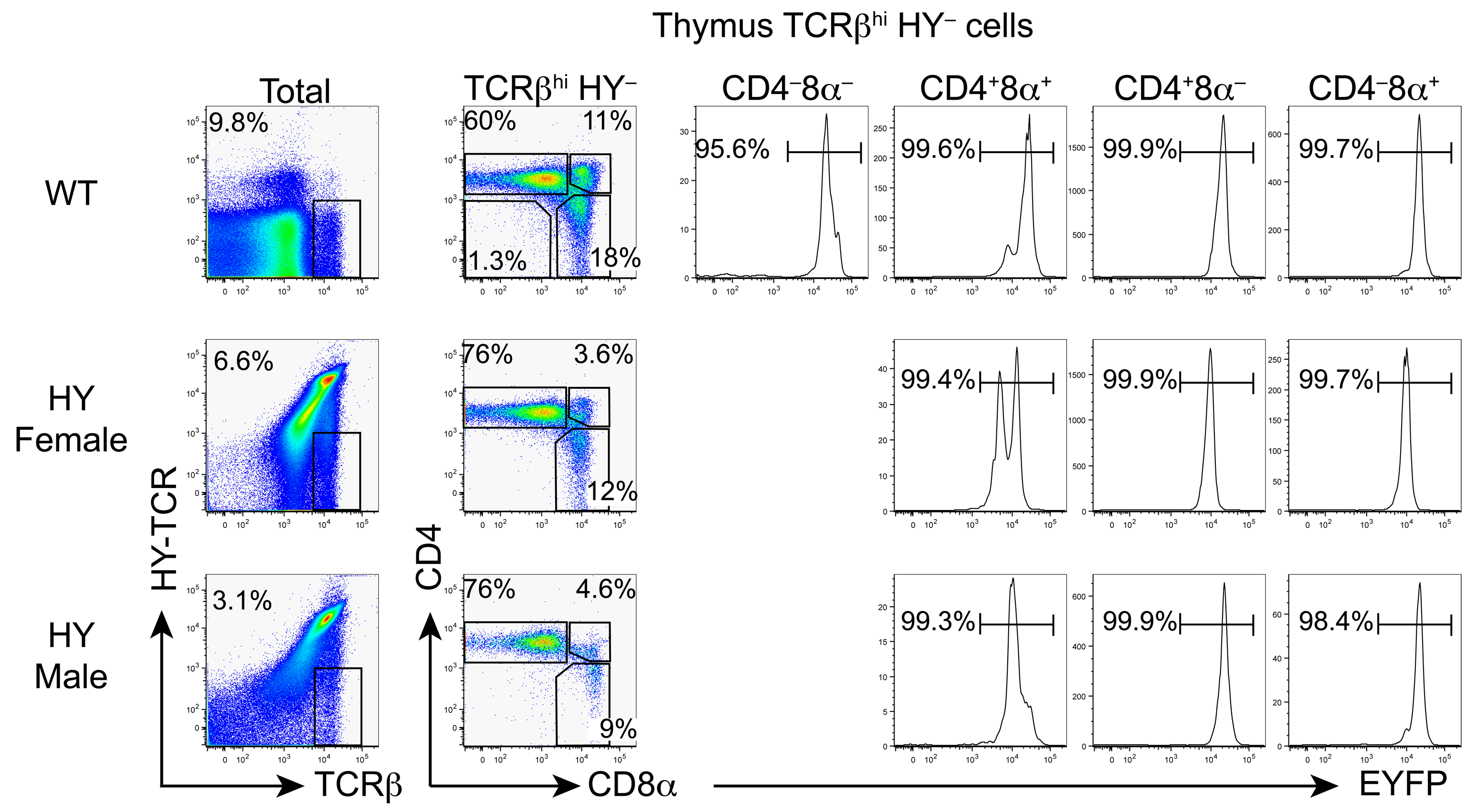

Supplement: Figure S1 — Fate-mapping of thymocytes from WT and HY-TCR transgenic mice expressing endogenous TCRα using RORγt. CD4 and CD8α expression by gated HY(T3.70)− TCRβhi thymocytes (left column) is shown in the second column. EYFP expression by HY−CD4−8α−, CD4+8α+, CD4+8α− and CD4−8α+subsets is shown. (There were very few, if any, HY− TCRβhi CD4−8− cells in the thymus of HY transgenic female or male mice.) (3.03 MB TIF) [file pone.0001512.s001.tif]

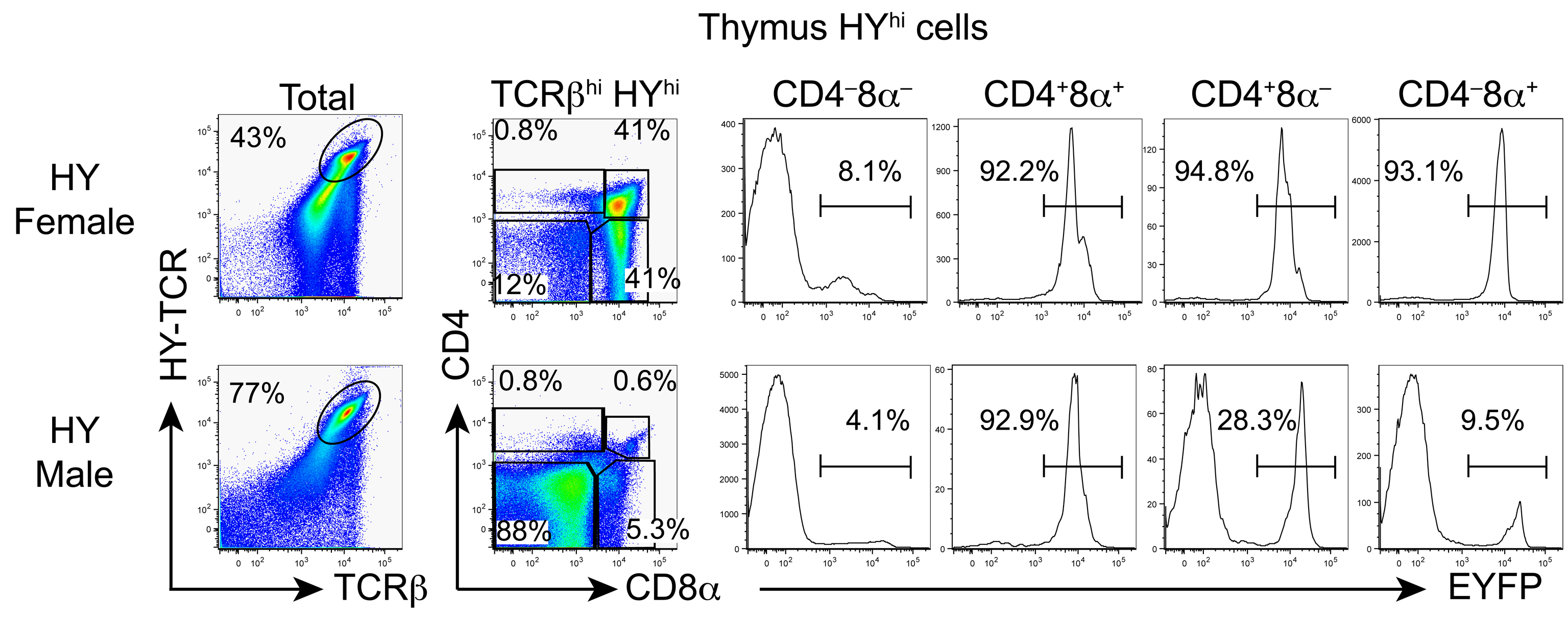

Supplement: Figure S2 — Fate mapping of thymocytes with the transgenic HY-TCR using RORγt-cre induced EYFP labeling. CD4 and CD8α expression by gated HY(T3.70)hi thymocytes (left column) is shown in the second column. EYFP expression by CD4−8α−, CD4+8α+, CD4+8α− and CD4−8α+ subsets is shown. (2.34 MB TIF) [file pone.0001512.s002.tif]

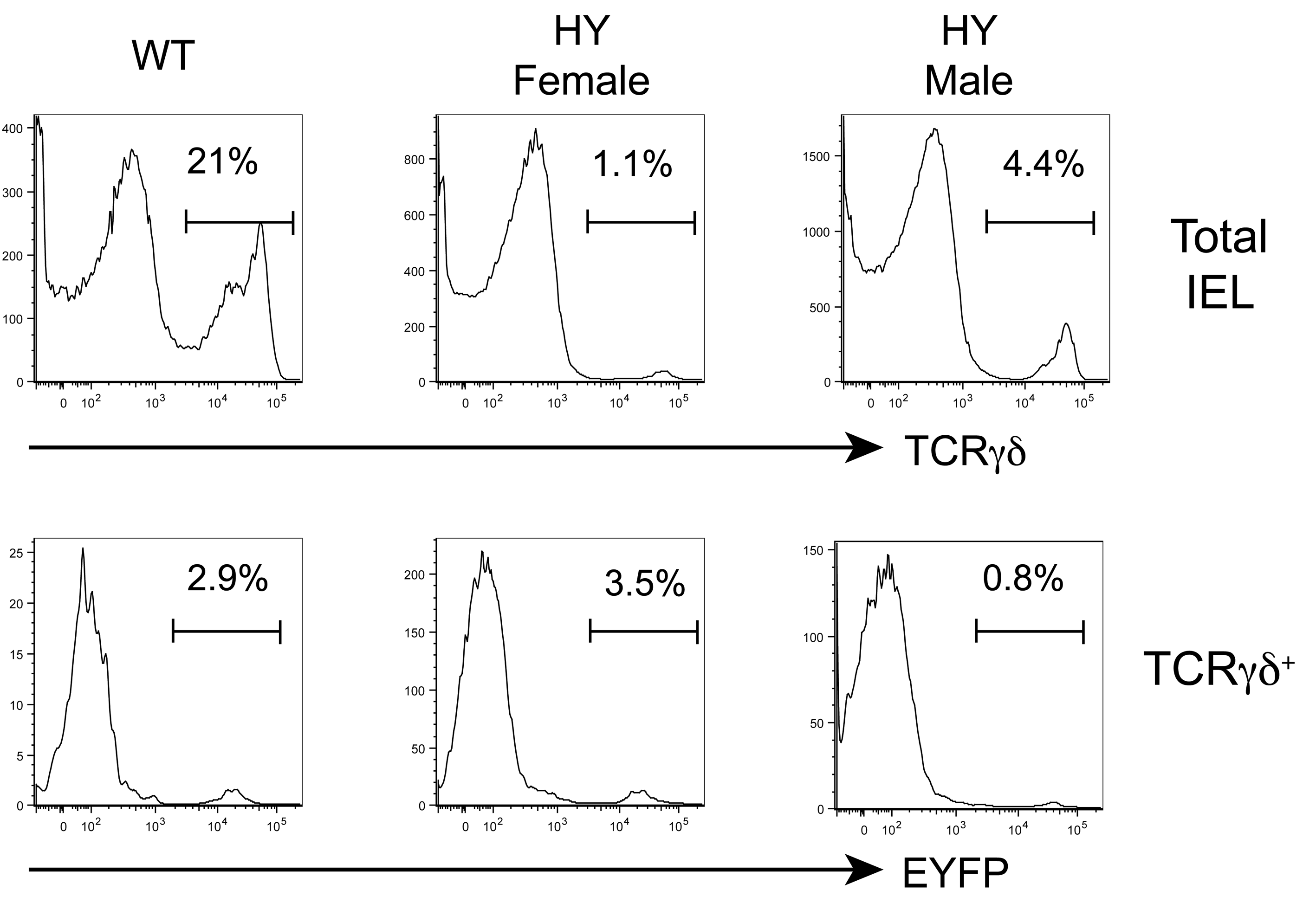

Supplement: Figure S3 — EYFP expression in TCRγδ IEL from RORγt-cre; ROSA26-stop-EYFP mice. TCRγδ positive IEL from WT and male and female HY-TCR transgenic mice were gated (top) and EYFP expression is shown (bottom). (0.69 MB TIF) [file pone.0001512.s003.tif]
